# Which Part of a Short, Global Risk Assessment, the Risk Instrument for Screening in the Community, Predicts Adverse Healthcare Outcomes?

**DOI:** 10.1155/2015/256414

**Published:** 2015-08-05

**Authors:** Rónán O'Caoimh, Carol FitzGerald, Una Cronin, Anton Svendrovski, Yang Gao, Elizabeth Healy, Elizabeth O'Connell, Gabrielle O'Keeffe, Eileen O'Herlihy, Elizabeth Weathers, Nicola Cornally, Patricia Leahy-Warren, Francesc Orfila, Constança Paúl, Roger Clarnette, D. William Molloy

**Affiliations:** ^1^Centre for Gerontology and Rehabilitation, University College Cork, St. Finbarr's Hospital, Douglas Road, Cork, Ireland; ^2^COLLAGE (Collaboration on Ageing), University College Cork, Cork City and Louth Age Friendly County Initiative, County Louth, Ireland; ^3^UZIK Consulting Inc., 86 Gerrard Street E, Unit 12D, Toronto, ON, Canada M5B 2J1; ^4^Centre for Public Health Nursing, Ballincollig and Bishopstown, County Cork, Ireland; ^5^Centre for Public Health Nursing, Mahon and Ballintemple, Cork, Ireland; ^6^Health Service Executive, South Lee, St. Finbarr's Hospital, Cork, Ireland; ^7^School of Nursing & Midwifery, University College Cork, Brookfield Health Sciences Complex, College Road, Cork, Ireland; ^8^Primary Healthcare University Research Institute, IDIAP Jordi Gol, Barcelona, Spain; ^9^UNIFAI/ICBAS, Institute of Biomedical Sciences Abel Salazar, University of Porto, 4050-313 Porto, Portugal; ^10^School of Medicine and Pharmacology, University of Western Australia, 35 Stirling Hwy, Crawley, WA 6009, Australia

## Abstract

The Risk Instrument for Screening in the Community (RISC) is a short, global risk assessment to identify community-dwelling older adults' one-year risk of institutionalisation, hospitalisation, and death. We investigated the contribution that the three components of the RISC (*concern*, its *severity*, and the ability of the *caregiver network* to manage concern) make to the accuracy of the instrument, across its three domains (mental state, activities of daily living (ADL), and medical state), by comparing their accuracy to other assessment instruments in the prospective Community Assessment of Risk and Treatment Strategies study. RISC scores were available for 782 patients. Across all three domains each subtest more accurately predicted institutionalisation compared to hospitalisation or death. The *caregiver network's* ability to manage ADL more accurately predicted institutionalisation (AUC 0.68) compared to hospitalisation (AUC 0.57, *P* = 0.01) or death (AUC 0.59, *P* = 0.046), comparing favourably with the Barthel Index (AUC 0.67). The *severity* of ADL (AUC 0.63), medical state (AUC 0.62), Clinical Frailty Scale (AUC 0.67), and Charlson Comorbidity Index (AUC 0.66) scores had similar accuracy in predicting mortality. Risk of hospitalisation was difficult to predict. Thus, each component, and particularly the *caregiver network*, had reasonable accuracy in predicting institutionalisation. No subtest or assessment instrument accurately predicted risk of hospitalisation.

## 1. Introduction

Population ageing [1] is associated with rising numbers of frail and functionally impaired community-dwelling older adults [2]. As time is limited in clinical practice, short risk prediction instruments are useful in identifying frailty [3] and quantifying the potential for adverse healthcare outcomes in this population [4]. Traditionally, healthcare practitioners have used demographic details and a battery of cognitive and functional tests in an attempt to predict risk [5], triage patients and rationalize the provision of limited healthcare resources [4]. Although multiple measures of frailty exist; few have been tested for reliability or validity [6].

More recently, instruments to identify specific adverse outcomes have been developed. These include tools to measure the likelihood of hospitalisation [7], readmission [8], institutionalisation [9], and mortality [10], often within a defined period of time. Few have targeted risk of institutionalisation, an important marker of healthcare utilisation [11]. Institutionalisation in turn is associated with other adverse outcomes such as risk of death [12]. Identifying risk is important as it singles out those who may benefit from more intensive, targeted interventions [4]. Few instruments are available in the community to screen large numbers of patients quickly and in their own environment for risk of functional decline, while simultaneously measuring risk of hospitalisation, institutionalisation, and death.

The Risk Instrument for Screening in the Community (RISC) is a new, short [13, 14], reliable [15], and valid [16] global subjective assessment of risk, designed for use by community healthcare workers. It was developed as part of Irelands' European Innovation Project on Active and Healthy Ageing three-star reference site the COLLaboration on AGEing (COLLAGE) [17]; see the Community Assessment of Risk and Treatment Strategies (CARTS) study at http://www.collage-ireland.eu/. The RISC identifies the presence and severity of concern in three domains (mental state, ADL, and medical state) [14]. Based upon the ability of an individuals' caregiver network to manage the patients' care needs, the one-year risk of three adverse outcomes, hospitalisation, institutionalisation, and death, is scored according to the magnitude and likelihood of an event, from 1 (minimal and rare) to 5 (extreme and certain). Short, often single, question screens similar to this have been used successfully in other studies. These include the “surprise question” [18], an independent predictor of one-year mortality, validated in different patient groupings [19], and the “Yale-Brown” [20] and “down-hearted and blue?” [21] single depression questions. Similarly, a single question “is the patient frail?” correlates with the Clinical Frailty Scale [13].

The contribution that each part of the RISC contributes to the overall predictive validity of the tool is unknown. Given this, we sought to investigate which part of the RISC contributes to the sensitivity and specificity of the instrument and how the components of the RISC compare to a traditional battery of screening instruments and to identify the components of the RISC which predict each of the three adverse healthcare outcomes under consideration.

## 2. Materials and Methods

### 2.1. The Risk Instrument for Screening in the Community (RISC)

The RISC collects demographic data and scores three domains: mental state, ADL (functional state), and medical state. Each has three components, called steps. The first step identifies whether there is a* concern* about this domain and is scored dichotomously (Yes/No). If there is no concern, the rater moves on to the next domain. If there is concern, the rater assesses the* severity* of the* concern* on a scale from one to three (mild, moderate, or severe). The ability of the* caregiver network* to manage the concern, within each domain, is then scored using a five-point Likert scale from 1 to 5 (1: can manage; 2: carer strain; 3: some gaps; 4: cannot manage; 5: absent/liability). The* caregiver network* includes the formal and informal resources and services that are available to the person. Finally, the* severity* of concern and the ability of the* caregiver network* are taken into account when completing the three global risk scores* of institutionalisation*,* hospitalisation,* and* death*, within one-year of assessment. These are also scored on a Likert scale from 1 (minimal/rare) to 5 (extremely likely/certain). In order to analyze the data, patients were subsequently divided into minimum (global risk score of 1 or 2) and maximum (global risk score of 3, 4, or 5) risk of each adverse healthcare outcome [14].

### 2.2. Patients

This study includes a secondary analysis of 803 patients included in the CARTS study. All patients were community-dwelling older adults, aged over 65 years. Only those recently reviewed and under long-term follow-up by their public health nurse (PHN) were included. The baseline characteristics of these patients have been published previously [13]. In summary, the median age of patients was 80 years (interquartile range 10) and 64% were females. Additional demographics and the results of a selection of cognitive and functional assessments were also available. The median Barthel Index (BI) score was 18 (+/−6), abbreviated mental test score (AMTS) [22] was ten (+/− <1), Charlson Comorbidity Index [23] score was one (+/− two), and the Clinical Frailty Scale (CFS) [24] was five (+/− two).

### 2.3. Data Collection and Sampling

The collection of data in the CARTS study has been described previously [13]. In summary, all PHN sectors in County Cork were invited to participate. Two, Ballincollig and Bishopstown and Mahon and Ballintemple, were the first respondents and were sampled based on the nonprobability method of convenience sampling using a quota method. All PHNs (*n* = 15) from these centres were trained and certified in scoring the RISC [15, 25]. Scoring was based on the PHNs knowledge of the patients. Each PHN only scored patients directly under their care. Demographic data were recorded from PHN records by a clinician blinded to the RISC scores. One year follow-up data on hospitalisation and death were obtained from the hospital in-patient enquiry system of all hospitals in Cork. Follow-up data on institutionalisation were obtained from the Cork Local Placement Forum. Ethical approval for the CARTS study was granted by the Clinical Research Ethics Committee of the Cork Teaching Hospitals and adhered to the tenants of Declaration of Helsinki.

### 2.4. Statistical Analysis

Data were analysed using SPSS version 20.0. Accuracy of components was determined from the AUC, calculated from receiver operating characteristic (ROC) curves. ROC scores above 0.50 indicated that the component had better predictive power than by chance alone. Nonoverlapping 95% confidence intervals (CI) suggested statistically significant differences between components. Cronbach's alpha coefficient (*α*) and the intraclass correlation coefficient (ICC) measured internal consistency.

## 3. Results

One-year outcomes comparing the RISC to the CFS have been presented previously [16]. In summary, RISC scores were available for 782 with 21 missing as patients were lost to follow-up. At baseline, 88%, 64%, and 79% were scored as minimum risk for institutionalisation, hospitalisation, and death, respectively. At one year, the incidence of the three adverse outcomes were 10.2% for institutionalisation, 17.7% for hospitalisation (at least one), and 15.6% for death. The accuracy of the global risk score to predict the outcomes was higher for risk of institutionalisation (AUC of 0.70) and death (AUC of 0.70) than hospitalisation (AUC of 0.61). Patients scored as maximum risk of institutionalisation had a 31.3% incidence of admission to long-term care compared with 7.1% for patients scored as minimum risk. Those scoring maximum RISC had a 25.4% and 33.5% incidence of hospitalisation and death compared to 13.2% and 10.8% for minimum risk patients, respectively.

Internal consistency between the three domains of the RISC, assessed with Cronbach's alpha, was high (*α* = 0.72) with scores above 0.7 indicating a high degree of reliability. The ICC, using one-way random effect model, was 0.68 (95% CI 0.61–0.70, *P* < 0.001), indicating a relatively high degree of agreement between the three domains of the RISC.

### 3.1. Components (Steps) of the RISC

Each step was examined to determine how accurately they predicted one-year outcomes. For the first step, assessing whether a* concern* was present or not, the AUC for predicting institutionalisation was 0.62 (95% CI: 0.55–0.69) for the mental state domain compared with 0.60 (95% CI: 0.54–0.66) for ADLs and 0.54 (95% CI: 0.48–0.61) for the medical state.* Concern* (Yes/No) on its own was a poor predictor of both hospitalisation and death, with all AUC values being less than 0.60 (see Table 1). The* severity* (mild, moderate, or severe) of each domain more accurately predicted institutionalisation compared to hospitalisation or death. The* severity* score of each domain was least accurate in predicting hospitalisation. The ability of the* caregiver network*, irrespective of domain assessed, more accurately predicted institutionalisation compared to the other adverse outcomes. The* caregiver networks'* ability to manage concern for ADL was significantly more accurate in predicting one-year risk of institutionalisation (AUC of 0.68, 95% CI: 0.62–0.74) compared to hospitalisation (AUC of 0.57, 95% CI: 0.52–0.63, *P* = 0.01) or death (AUC of 0.59, 95% CI: 0.53–0.65, *P* = 0.046). The* caregiver networks*' ability to manage mental and medical states predicted institutionalisation better than hospitalisation or death.

**Figure 1:**
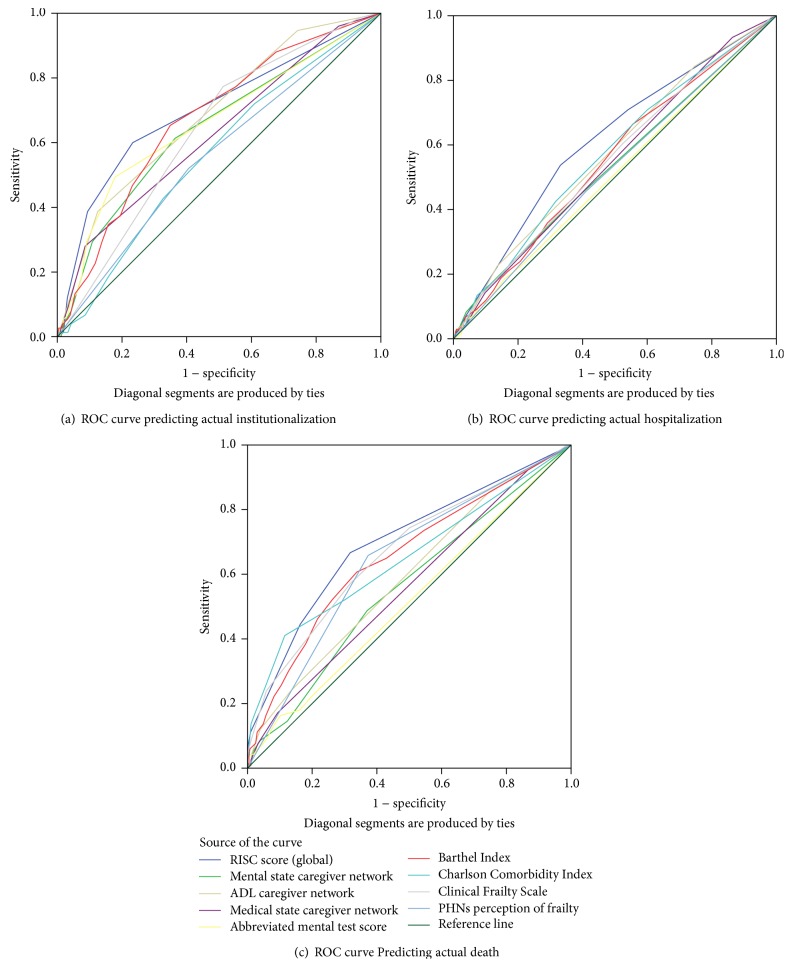
Receiver operating characteristic curves demonstrating the accuracy of the Risk Instrument for Screening in the Community (RISC), mental state, activities of daily living (ADL) and medical state domains, the abbreviated mental test score (AMTS), Barthel Index, Charlson Comorbidity Index, Clinical Frailty Scale, and public health nurses' (PHNs) perception of frailty in identifying one-year risk of (a) institutionalisation, (b) hospital admission (at least one), and (c) death.

The AUC values for the global RISC scores, the caregiver components of the RISC, the CFS, the PHNs perception of frailty, and a battery of cognitive and functional instruments routinely collected by PHNs are presented in Table 2 and Figure 1. The global RISC scores most accurately predicted all three adverse outcomes. The* caregiver networks'* ability to manage ADLs (AUC of 0.68) had similar accuracy in identifying one-year risk of institutionalisation as the BI (AUC of 0.67, *P* = 0.84), the AMTS (AUC of 0.66, *P* = 0.68), and the CFS (AUC of 0.63, *P* = 0.30) but was significantly more accurate than the Charlson Comorbidity Index (AUC of 0.55, *P* < 0.01). The BI was significantly more accurate in predicting institutionalisation (AUC of 0.67 95% CI: 0.61–0.73, *P* = 0.04) and death (AUC of 0.65 95% CI: 0.60–0.71, *P* ≤ 0.05) than hospitalisation (AUC of 0.58 95% CI: 0.50–0.61). The Charlson Comorbidity Index was a significantly more accurate predictor of death at one year (AUC of 0.66) than hospitalisation (AUC of 0.57), *P* = 0.02. The AMTS was only accurate for predicting institutionalisation (AUC of 0.66).

## 4. Discussion

This study describes the relationship between the components of the RISC across its three domains (mental state, ADL, and medical state), their accuracy in predicting the incidence of three adverse outcomes (institutionalisation, hospitalisation, and death), and the contribution that the components of the RISC provide in predicting each adverse healthcare outcome within one year of assessment, compared with a battery of assessment instruments, in a sample of community-dwelling older adults.

The results suggest that all components of the RISC were better able to predict institutionalisation than hospitalisation and death. The perceived ability of the* caregiver network* to manage patients' ADL was most accurate in predicting institutionalisation and hospitalisation. This component contributes much to the predictive power of the instrument as a whole. This would be expected, particularly for institutionalisation, as patients' social and caregiver networks play an important role in the mental [26] and physical health [27] of community-dwelling older adults. Indeed, caregiver burden is an established risk factor for all three adverse outcomes evaluated in this study [28].

The overall accuracy of the RISC subtests for predicting hospitalisation, within one year of the assessment, was poor. Only the perceived* severity* of concern of a patients' medical state and the ability of an individuals'* caregiver network* to manage a persons' ADLs had some, albeit weak (AUC < 0.60), ability to predict the one-year rate of hospitalisation. This difficulty in predicting hospitalisation was seen in the validation of the RISC [16] and may reflect the complexity associated with predicting hospital admission in such a frail population (median Clinical Frailty Scale score of 5/9). Several studies and instruments have been developed to predict readmission to hospital, many with poor accuracy [8]. The Hospital Admission Risk Profile, for example, stratifies patients into low, medium, and high risk based on three factors: age, cognitive function, and preadmission ADL function, with an AUC of 0.65 for predicting hospitalisation [29]. In part, this may relate to the fact that short risk prediction instruments fail to incorporate other complex factors influencing hospitalisation including system-specific and patient-specific factors [8]. While the RISC was designed to measure risk within one year, most instruments measure risk of hospitalisation or readmission within a short period such as 30 days [8]. Available studies predicting outcomes over longer periods, up to one year, have found similar albeit higher accuracy [30]. Given that predictors of short- and long-term adverse healthcare outcomes are likely to be different [31], the accuracy of the RISC in predicting hospitalisation within one year seems reasonable. The accuracy of the RISC for predicting one-year mortality was superior to hospitalisation. This was consistent with other studies [32, 33] including those incorporating multidimensional interdisciplinary CGA, which is a better predictor of one-year mortality than comorbidity or prognostic indices [34]. The reasons for this are complex and unclear. The factors predicting hospitalisation and hospital readmission are more complex than those predicting death and include hospital and healthcare system-level factors which are often location specific and cannot easily be incorporated into short and generalizable risk prediction models [8, 33]. Further, while these outcomes are not mutually exclusive, not all hospitalisations result in death and vice versa.

Comparing the accuracy of the other instruments used in the CARTS study to the components of the RISC suggests that the* caregiver network* component of the RISC (ADLs) had comparable accuracy in identifying risk of institutionalisation as the Clinical Frailty Scale, a marker of frailty. Likewise, both had similar accuracy for one-year mortality. The Charlson Comorbidity Index, a well-validated measure of comorbidity studied across a wide variety of clinical settings, was better able to predict mortality than hospitalisation. This is similar to the Cumulative Illness Rating Scale [35], another measure of comorbidity, which is more accurate at predicting institutionalisation than death [24]. The BI, a measure of ADL had similar accuracy to a measure of cognition (AMTS) in predicting institutionalisation. This would be expected given that function is important in determining nursing home placement in patients with [36] and without cognitive impairment [37]. This was mirrored in the greater accuracy of the* caregiver networks'* ability to manage ADL over its ability to manage patients' mental state.

The strengths of this study are the large numbers of community-dwelling older adults included and the comprehensive nature of the PHN records and that the instrument was validated in a sample of urban-suburban community-dwelling older adults in busy health centres. Another strength is that this tool employs a simple Likert scale to score the ability of the caregiver network to manage concerns in three domains. To our knowledge, there is no other instrument that measures the caregiver network in this way. This study does have limitations. Collection of demographic data was based upon a retrospective review of the patients' PHN records. Furthermore, the sample only included patients under active follow-up by their PHN, which may have created selection bias. Likewise, patients were predominantly functionally independent (median BI score of 18), cognitively intact (median AMTS score 10), and without significant comorbidity (median Charlson Comorbidity Index score of one), which may also have contributed to this bias. However, most patients were mildly frail as judged by the CFS (median score of five). Future studies should investigate the use of the RISC in other populations including those not under PHN surveillance and those more or less frail than this current sample. As the RISC is designed to detect outcomes within one year of assessment, the “predictive window” in this study differs from other studies, reducing the ability to directly compare outcomes and predictive validity. Likewise, the performances of many of the RISC subtests were low with AUCs at best between 0.6 and 0.7. This generally indicates low accuracy in correctly identifying outcomes [38]. That said, the objective of this study was to compare a lengthy battery of assessment instruments with a single, short subjective screen for risk. The accuracy of the test compares favourably with the other instruments included in this study and in other published papers.

## 5. Conclusion

In conclusion, the ability to identify high-risk individuals constitutes the first step in any strategy to target vulnerable, frail patients. The RISC is a short, reliable, and validated risk prediction screen for use in the community to identify risk of adverse healthcare outcomes. The RISC is similar to other short screening instruments like the Clinical Frailty Scale, the application of which, like the RISC, requires judgment and a degree of subjectivity [24]. In this study, the most accurate RISC subtest for institutionalisation was the ADL* caregiver network*. The most accurate subtest for death was ADL* severity*. The measurements of internal consistency suggest that all components of the RISC contribute to the overall predictive validity of the instrument. The RISC performed better than a selection of other assessment instruments, routinely collected by PHNs, in this sample of community-dwelling older adults. Further research is now required to compare the RISC with other validated risk tools, single-question screens, frailty measures, and comprehensive assessment instruments such as the InterRAI [39].

## Figures and Tables

**Table 1:** Receiver operating characteristic (ROC) curve area under the curve scores and 95% confidence intervals (CI) for the global risk score and components of the Risk Instrument for Screening in the Community (RISC) scores including mental state, activities of daily living (ADL), and medical state domains, the primary caregiver, and primary cohabitant (who the patient is living with), for predicting one-year risk of institutionalisation, hospitalisation, and death.

Variable	Actual outcomes		
	Institutionalization	Hospitalization	Death
RISC global risk score (CI)	0.70 (0.62–0.76)^***^	0.61 (0.55–0.66)^***^	0.70 (0.64–0.75)^***^
Mental state			
Mental state *concern *	0.62 (0.55–0.69)^***^	0.52 (0.47–0.58)	0.56 (0.50–0.61)^*^
Mental state *severity* of concern	0.64 (0.57–0.71)^***^	0.53 (0.47–0.58)	0.56 (0.51–0.62)^*^
Mental state *caregiver network *	0.64 (0.57–0.71)^***^	0.53 (0.47–0.58)	0.56 (0.50–0.61)
ADLs			
ADLs *concern *	0.60 (0.54–0.66)^**^	0.55 (0.50–0.60)	0.56 (0.50–0.61)^*^
ADLs *severity* of concern	0.66 (0.60–0.72)^***^	0.54 (0.49–0.59)^*^	0.63 (0.58–0.69)^***^
ADLs *caregiver network *	0.68 (0.62–0.74)^***^	0.57 (0.52–0.63)^**^	0.59 (0.53–0.65)^**^
Medical state			
Medical state *concern *	0.54 (0.48–0.61)	0.52 (0.47–0.58)	0.53 (0.48–0.59)
Medical state *severity* of concern	0.62 (0.55–0.69)^***^	0.57 (0.52–0.62)^*^	0.62 (0.56–0.67)^***^
Medical state *caregiver network *	0.63 (0.56–0.69)^***^	0.54 (0.49–0.59)	0.56 (0.50–0.61)^*^

^∗^Statistically significant with *P* value <0.05.

^**^Statistically significant with *P* value <0.01.

^***^Statistically significant with *P* value <0.001.

**Table 2:** Comparison of the accuracy and area under the curve (AUC) scores with 95% confidence intervals (CI), of the Risk Instrument for Screening in the Community (RISC), the caregiver network for each domain and a selection of cognitive and functional tests including the Barthel Index, abbreviated mental test score, Charlson Comorbidity Index, and the Clinical Frailty Score.

Variable	Institutionalization	Hospitalization	Death
	AUC (95% CI)	AUC (95% CI)	AUC (95% CI)
RISC global risk score	0.70 (0.62–0.76)^***^	0.61 (0.55–0.66)^***^	0.70 (0.64–0.75)^***^
Mental state *caregiver network *	0.64 (0.57–0.71)^***^	0.53 (0.47–0.58)	0.56 (0.50–0.61)
ADL *caregiver network *	0.68 (0.62–0.74)^***^	0.57 (0.52–0.63)^**^	0.59 (0.53–0.65)^**^
Medical state *caregiver network *	0.63 (0.56–0.69)^***^	0.54 (0.49–0.59)	0.56 (0.50–0.61)^*^
Barthel Index	0.67 (0.61–0.73)^***^	0.58 (0.50–0.61)^*^	0.65 (0.60–0.71)^***^
Abbreviated mental test score	0.66 (0.59–0.73)^***^	0.51 (0.46–0.56)	0.51 (0.46–0.57)
Charlson Comorbidity Index	0.55 (0.49–0.62)	0.57 (0.52–0.62)^**^	0.66 (0.60–0.72)^***^
Clinical Frailty Scale	0.63 (0.57–0.67)^***^	0.55 (0.50–0.61)^*^	0.67 (0.61–0.72)^***^
PHNs perception of frailty	0.56 (0.49–0.62)	0.53 (0.47–0.58)	0.64 (0.59–0.70)^***^

^∗^Statistically significant with *P* value <0.05.

^**^Statistically significant with *P* value <0.01.

^***^Statistically significant with *P* value <0.001.
